# Co-implementation: collaborative and concurrent approaches to advance embedded implementation in the health sciences

**DOI:** 10.3389/frhs.2023.1068297

**Published:** 2023-12-20

**Authors:** Mandy M. Archibald

**Affiliations:** Helen Glass Centre for Nursing, University of Manitoba, Winnipeg, MB, Canada

**Keywords:** co-implementation, embedded implementation, embedded research, co-design, learning health systems

## Abstract

There is a global movement towards stakeholder engagement in healthcare research. This movement has been catalyzed by a need to create context relevant evidence of maximal utility to health service provision and policy. The concept of “co-implementation” has potential to inform and extend these discussions of partnership and to complement the growing literature on collaborative implementation. Attending to this concept may preempt conceptual confusion and provide opportunities for sustainable and context-responsive embedded research necessary for the strengthening of health systems. In this perspective article, I seek to advance the discussion of co-implementation through an examination of the concept and through consideration of it merits to the health sciences.

## 1. Introduction

The landscape of implementation science is changing, with partnered and co-designed approaches gaining recognition in a global movement of stakeholder engagement (1, 2). In part, this movement towards collaboration in implementation has been motivated by the realization that interventions effective in one setting may not be effective in another setting, due to the profound influence of context on intervention outcomes (3). Ensuring an intervention fits with local context in terms of its acceptability, feasibility, affordability, and congruence with culture and priorities, is paramount to the field of implementation science, wherein the primary concern is application in the messiness of real-world settings (4). “Fit” it seems, is best negotiated through dialogical approaches enabled through partnership between those key actors invested in the process and outcomes of implementation.

The concept of partnership then, has grown exponentially within the implementation science literature. Implementation science involves the scientific study of approaches (i.e., methods and strategies) aimed at facilitating the use of research and evidence-based practices into regular use. Partnership within implementation science involves various iterations of related, blurry, and often conflated concepts such as co-production (i.e., an umbrella term identifying stakeholder input into service delivery), co-design (i.e., collective working together across the design process), and co-creation (i.e., developing a shared body of useable knowledge, often for the purpose of innovation), which are differentially conceptualized and operationalized (5–7). Diverse understandings and applications of these terms has resulted in a complex definitional landscape and an emphasis on the underlying values of the respective terms (8).

Recently, our partnered work with a youth rehabilitation center gave rise to interest in the co-implementation of research within the routine operations and infrastructure of the facility. This prompted the question of whether existing terms would be helpful in informing implementation, or whether the concept of co-implementation itself has potential to inform and extend this discussion of partnership in implementation research. Attending to co-implementation as a concept may provide opportunities for its application to the complex problems of interest to the health sciences. In this perspective article, I explore co-implementation as a concept and consider its merits to the health sciences.

## 2. Co-design in implementation research: how engaged are stakeholders, anyways?

The movement towards co-design has been catalyzed by awareness of research waste. A staggering volume of dollars is invested in medical research that is subsequently not published, inadequately reported, or conducted within suboptimal design (7). Within this context, Slattery et al. (7) identified that a lack of awareness of stakeholder priorities in the conduct of research as another key contributor to research waste. The premise for this argument is that research is being conducted that is of limited value to knowledge users. As such, stakeholder involvement will improve research relevance and use, leading to improved service delivery and health outcomes (9, 10). This premise is widely accepted by researchers within participatory research environments who recognize the merits of stakeholder involvement in health research. However, co-design as a participatory concept reflects a continuum of stakeholder involvement that problematically may fall short of informing the implementation process.

The extent of stakeholder involvement in research is project dependent, with some projects emphasizing integrated involvement (e.g., from research conceptualization) with others benefiting from periodic consultation (e.g., advisement on data collection approaches). Often, assessing the meaningfulness of stakeholder involvement is hindered by a need for more thorough reporting (8). Yet research demonstrates that the timing and extent of engagement (e.g., the phase that stakeholders are involved) differs in accordance with the approach used, with stakeholder engagement emphasizing the interpretation of findings and dissemination; patient engagement involving the application of knowledge, and participatory methods emphasizing involvement in the design and conduct of research (7). Further, studies reporting on stakeholder involvement demonstrate limitations to the extent of their involvement; further work is needed to conduct research aligned with stakeholder priorities (11).

Slattery et al. (7) also examined the activities that stakeholders were involved in, with focus group, interviews, surveys and rating methods being the most common. The extent of engagement differed greatly between studies. Critically, co-design with practitioner groups received little description in the included studies despite frequent mention of these groups as stakeholders. These shortcomings reflect concerns espoused by scholars that the “core values underpinning coproduction and co-design may be being diminished” (8). This remains problematic in the context of implementation wherein meaningful stakeholder involvement is foundational to establishing contextual fit and promoting sustainability.

There is inherent utility in applying the participatory zeitgeist (12) towards implementation, yet, nuances in terminological definitions and applications raise questions regarding the appropriateness of the widely used “co” terms in the context of implementation. The literature suggests that the application of co-design or co-creation principles within implementation is comparatively lacking, despite emergence of relatively new frameworks aimed at supporting partnered quality improvement and evidence based practice initiatives within health services (e.g., Queri roadmap) (13). Peters et al. (14) highlight such shortcomings in their review of evidence based guideline implementation, finding that only 35.6% of studies employed some component of stakeholder engagement. Of these, the majority were poorly reported, rendering it difficult to assess the extent and utility of stakeholder engagement. Overlooking the critical input of stakeholders and specifically, implementers, in the planning and execution of implementation processes, problematically risks overlooking their insider knowledge and insights into prospective barriers to implementation (15). While engaging stakeholders in earlier research stages is dominant in overviews of co-design literature, co-implementation as a process has not fully benefited from the advancements noted in the co-design and participatory health research discourses. What then, is co-implementation, and what could this concept offer for the future of implementation science in the health sciences?

## 3. Co- implementation: the what, the where and the why

The term co-implementation appears to be relatively new, but is situated within a growing body of literature discussing collaboration in implementation. A search of co-implementation within peer reviewed articles with no date limiters conducted in September 2022 retrieved 4 records in CINAHL, 21 records in Pubmed, and 48 records within the SCOPUS database, respectively. Of these, 13 were assessed as relevant to the co-implementation concept (e.g., discussed co-implementation or operationalized the concept in a meaningful way) within healthcare. The other articles—gathered to inform a multidisciplinary understanding of the concept—reflected civic and environmental applications. Publication dates ranged from 2008 to 2022, with over half (55%) published within the last 3 years (2019–2022).

Divergent development of the co-implementation concept was noted across disciplines and domains of application. Numerically, this can be seen in the distribution of articles examining or applying co-implementation within the health sciences. However, authors reported various levels of development and application of stakeholder engaged implementation approaches in their respective fields of study. For example, Shackleton et al. (16) reported that despite growing interest into stakeholder engagement in invasion science, few published reports use active engagement strategies with multiple stakeholder groups, which limits benefit given that no two-way flow of knowledge is achieved. Within healthcare, the readership of such dedicated journals as *Implementation Science*, and academic champions of implementation science in health care have made progress towards more meaningful engagement, often through the development of frameworks but also through growing emphasis on co-design and co-creation.

Analysis of this small but representative body of literature revealed two main narratives of co-implementation. The first is that co-implementation involves the simultaneous implementation of programs or initiatives, often within an existing service delivery model. Here, “co” refers to the “concurrent” implementation of services. The second perspective regards co-implementation as an extension of co-design. Here, “co” refers to “collaborative” input into the implementation of an initiative. These two iterations of co-implementation carry distinct contributions to the conversation on advancing participation and reducing waste in health research, while also thematically overlapping in worthwhile ways.

For instance, regarding the co-implementation of services within the “co as concurrent” model, Bhutta et al. (14) conducted a review to assess the effectiveness of co-implementing interventions through existing community-based programs. Questioning the merits of integrated vs. non-integrated (or stand-alone) program delivery, they identified that co-implementation within well-resourced community-based programs offer potential to scale up interventions for infectious diseases of poverty. Bhutta et al. (17) posit that integrated co-implementation is the more feasible and cost-effective option when the target condition is endemic to the area of service provision. By highlighting the merits of school-based delivery for their study context, the authors also highlight a key possible benefit of co-implementation: no additional staff would be required once existing staff are adequately trained in implementation strategy. They conclude that investment in service delivery capacity is paramount to reducing barriers to implementation that could thwart sustainability or retention. Similarly, Amazigo (18) discussed the co-implementation of an onchocerciasis control program within other community-based health service delivery models. They identified this concurrent implementation and intervention embeddedness as a key aspect of the program's effect; it improved the therapeutic coverage for onchocerciasis while concurrently improving service delivery in other areas with the greatest gains seen in vaccination programming. Their approach, as well as others employing co-implementation in this manner, also necessitates community participation and involvement, suggesting a “co as collaborative” component as well.

The concurrent model of co-implementation provides important insights into the possibilities of embeddedness in research. This is strengthened through the possible symbiosis of mutually informing knowledge production and implementation occurring through collaboration. Literature within the “co as collaborative” model provides such insight, and reflects a complexity based view of health systems wherein the roles of actors, pathways and associated attributes, such as trust, are emphasized (6). Complex systems consist of networked webs of relationships between various stakeholders who self-organize through interactions, which in turn enables learning, problem-solving, and the development of shared knowledge (6). Such interactions create a mediating effect towards the production and use of evidence, recasting the research-practice “gap” as an open ecosystem comprised of adaptive and interactive networks (6, 19, 20).

Despite differences in each framing of co-implementation, dominant thematic overlaps are notable in the literature and reflect a logic structure pertaining to the benefits and possibilities of co-implementation. These include: (i) co-implementation integrates interventions within regular service delivery (i.e., concurrent implementation), (ii) successfully achieving this integration requires attention to power structures, networks, and democratizing relationships, (iii) integration of interventions and services requires attention to diverse indicators, or outcomes, (iv) identifying these outcomes and readiness for integration requires collaboration (i.e., collaborative implementation), which leads to (v) co-implementation as an embedded approach (i.e., embedded implementation).

### 3.1. Thematic considerations regarding co-implementation

#### 3.1.1. Integration within regular service delivery

Highly cited frameworks guiding translation and implementation, such as Reach, Effectiveness, Adoption, Implementation, and Maintenance (RE-AIM), “T” model, and Knowledge to Action Framework (KTA), position research and context (i.e., implementation locale) as two points to be bridged, the gap between them mainly governed by “time”—time for research to be used to inform practice or policy, “lag-time” (9). Conversely, integrating interventions within service delivery eliminates this gap, thereby shifting the conceptual model towards iterative feedback and improvement loops entrenched within a complex open system. The integration of interventions within regular service delivery is advocated when services are well resourced, and the benefits of intervention integration can be maximized by alignment with the serviced population (21). Integrating the “collaborative co” along with the “concurrent co” using guiding frameworks for collaboration as well as leveraging the capacity building required of this approach would facilitate seamless integration and identification of strategies, as well as future areas of focus, for integrated implementation initiatives. A necessary accompaniment to this integration is the use of evaluation metrics associated with implementation; routinizing their collection is a paramount consideration related to sustainability and to minimizing workflow disruptions (21).

#### 3.1.2. Attention to power structures and democratizing relationships

Co-design predicates that democratic partnerships can be developed between community stakeholders and researchers with the objective that end users will be involved in the entirety of the research process, extending from design through to the uptake of the research findings (22). Despite its apparent clarity, involving stakeholders in research through co-design is not homogenous; the nature, purpose and timing of engagement exerts notable effects on the power-distribution occurring within the team dynamic. To this point, Paidakaki et al. (23) discusses the role of “alternative co-producers” as “important political and institutional actors in co-implementation processes” (p.2). In their context of equitable housing, these alternative co-producers are “pro-equity and pro-co materializing non-profit housing policy implementers” (p.2). The emphasis on pro-equity reflects the orientation of implementation science as emphasizing “intention to reach” rather than “intention to treat” (4). In their context, alternative co-producers are in unique positions to draw empowerment from their grassroots foundations and exert their influence at negotiation tables. Paidakaki et al. (23) then introduces the concept of “co-implementation” as critical for strengthening political agency and “expanding the meaning and usefulness of co-production in planning theory and practice” (p. 2).

Establishing trust and pathways or channels between stakeholders in complex systems are critical to the flow of evidence and to authentic collaborative communication (6). Similarly, transdisciplinary environments involved in knowledge translation require strong leadership (24) but workplace democratization is also a necessary condition. Just as the knowledge translation discourse is increasingly looking towards the organization rather than solely emphasizing individuals' responsibility for research use (25), so too must discussions of democratization consider workplace structure. In this regard, the Democratizing Work Manifesto attends to the disempowerment of workers that results from inequitable power distribution, arguing that workers should have right to participate more substantively in workplace governance and decision making (26). The disempowerment resulting from a lack of democratic participation reduces participation, and undermines the creativity and innovation benefits offered by workers innovation (26). A movement towards co-implementation hence requires attention to democratic relationships but also to the structure of workplace organization that enables such relationships to occur.

#### 3.1.3. Attention to diverse indicators and outcomes

Indicators and outcomes for co-implementation should reflect the mechanisms (underlying processes that operate in specific contexts to produce an effect) (27) that contribute to the success of implementation and standard care integration, as well as components pertinent to the end points of care. For instance, research capacity and research resource investment are critical to achieving embedded research practices, as well as workplace satisfaction in workplaces where research practice is an expectation (28). Collecting data on health outcomes pertinent to interventions as well as routine service delivery are critical, and ideally should be included in research infrastructure—included data collection systems integrated with standard care as much as possible—using electronic health records (21).

Literature on co-implementation outside of the health sector provides additional insight into the diverse indicators and outcomes in consideration. For instance, the success of co-implementation, wherein health service outcomes as well as research outcomes are of interest, and wherein social and organizational factors as well as health resource factors impact success, mandates dialogic considerations of indicators. Social process indicators influencing governance of river basins for instance, focus on aspects of collaborative governance or deliberative democracy, since governance is seen as the primary failure of river basin management (29). Yet these social process indicators should be considered in light of quantifiable environmental outcome targets, since these exist in a synergistic and co-evolving system. Similarly, co-implementation as an embedded research-health service delivery model should include systems to capture data on practice, policy, and workflow, implementation metrics as well as the health impacts of interventions in relation to standard care (21).

#### 3.1.4. Collaborative implementation

“Co as collaborative” implementation was the second conception of co-implementation discussed, and positioned co-implementation as an extension of the concept of co-design. While this perspective emphasizes the collaborative working relationships of academics and stakeholders in the production of knowledge, it does not capture the possible symbiosis of mutually informing knowledge production and implementation possible within a democratized work context. Within this perspective, co-implementation should be viewed in tandem with 3 other processes associated with co-creation of knowledge. These include co-ideation, wherein the problem and possible solutions to the problem are jointly discussed; co-design, wherein the technical aspects of approaching the problem—such as the methods used—are collaboratively considered; and co-evaluation, wherein data collection is formally embedded in to the co-implementation process (9). To this point, Metz (6) highlights the importance of role clarity as stakeholders self-organize, reflecting the importance of networks and the ongoing adaptation influencing implementation within complex health systems.

#### 3.1.5. Co-Implementation as an embedded research approach

Embedded research is an inherently pragmatic approach that can involve the testing and subsequently smooth integration of interventions into existing work flows, using standard care and routinely collected measures (21, 30). While “embedded” can refer to different practices including embedding researchers in health service delivery settings to embedding research in policy processes (31) here, the under acknowledged area of “embedding research within practice through decision-maker-led partnerships” (p. ii99) is emphasized. This involves the use of research processes and metrics that are built into the data capture and existing workflows of a system, and into the routine delivery of health services. Embedded research is entwined with context-dependency, enabling continual feedback of context specific priorities with routinized data collection enabling insight into benefits at patient, client and organization levels. Critically, this form of context-specific evidence is what the World Health Organization regards as essential to informing policy and to strengthening health systems worldwide (32). Co-implementation –conceptualized as a collaborative *and* concurrent approach to implementation—is highly approach aligned with the ethos of embedded research.

## 4. Discussion

The values of co-design are becoming entrenched in academic healthcare dialogues. This movement is occurring within a context of mounting health system pressures related to increased service delivery needs, circumstances of austerity and financial uncertainty, and the establishment of an accountability paradigm surrounding health research. That health research is used to inform practice and policy is now the expectation. Despite the proliferation of frameworks and scientific growth in this area, there remains much opportunity to renew how the research-practice gap—including whether it is or should be a gap at all—is conceptualized and addressed.

Some scholars in this area have highlighted a lack of empirical evidence regarding whether and how co-design discussions have infiltrated the research and practice arenas. For example, Pearce et al. (9) examined how multisectoral collaborations occur within the field of suicide prevention. Multisectoral collaborations provide ideal cases to examine the processes of co-implementation, since they are inherently stakeholder engaged and “bottom-up participatory processes involving partnership between government, third-sector organizations, community members, citizens, and researchers to address social issues” (9) (p. 2). Frameworks emphasizing participation and integration (such as integrated knowledge translation for instance) commonly inform such efforts, with the level of equality achieved reflecting contingency on the approaches used (e.g., consultation vs. co-researching) (9).

Here, key characteristics and considerations related to co-implementation as a practice integrating concurrent and collaborative approaches have been offered. Among these is that co-implementation should be a democratic process, imbued with capacity building of co-researchers, in order to generate context specific evidence and practices of most use to health decision makers. Implementation science as an inherently interdisciplinary process informed by a multitude of theories adding to its richness will continue to benefit from broad reading of theorizing and application to inform insights into critical processes such as the intersection of governance and management, for instance, which influence how co-implementation would occur and be sustained in health systems. A renewed theoretical framework informed by co-implementation and drawing from related principles of embedded research and embedded implementation could amend characterizations of research and practice as a gap that needs bridging. Researchers are invited to extend the concepts offered here, and build upon co-implementation as a concept reflective of two main discourses identified in the literature– that of “co” as concurrent, wherein co-implementation involves the simultaneous implementation of programs or initiatives within existing service delivery models, and (“co” as collaborative, wherein co-implementation is an extension of co-design, requiring collaborative input into the implementation of initiatives. The thematic overlaps identified between these concepts suggest a logic structure pertaining to the benefits and possibilities of co-implementation. These include: (i) co-implementation integrates interventions within regular service delivery (i.e., concurrent implementation), (ii) successfully achieving this integration requires attention to power structures, networks, and democratizing relationships, (iii) integration of interventions and services requires attention to diverse indicators, or outcomes, (iv) identifying these outcomes and readiness for integration requires collaboration which leads to (v) co-implementation as an embedded approach (i.e., embedded implementation). This perspective offers a re-framing for health researchers, practitioners and systems interested in reducing research waste and supporting integration of research and practice in health systems.

## Data Availability

The original contributions presented in the study are included in the article/Supplementary Material, further inquiries can be directed to the corresponding author.
